# Regulatory dynamics distinguishing desiccation tolerance strategies within resurrection grasses

**DOI:** 10.1002/pld3.457

**Published:** 2022-12-13

**Authors:** Brian St. Aubin, Ching Man Wai, Sunil K. Kenchanmane Raju, Chad E. Niederhuth, Robert VanBuren

**Affiliations:** ^1^ Department of Horticulture Michigan State University East Lansing MI USA; ^2^ Plant Resilience Institute Michigan State University East Lansing MI USA; ^3^ Department of Plant Biology Michigan State University East Lansing MI USA

## Abstract

Desiccation tolerance has evolved recurrently in grasses using two unique strategies of either protecting or dismantling the photosynthetic apparatus to minimize photooxidative damage under life without water (anhydrobiosis). Here, we surveyed chromatin architecture and gene expression during desiccation in two closely related grasses with distinguishing desiccation tolerance strategies to identify regulatory dynamics underlying these unique adaptations. In both grasses, we observed a strong association between nearby chromatin accessibility and gene expression in desiccated tissues compared to well‐watered, reflecting an unusual chromatin stability under anhydrobiosis. Integration of chromatin accessibility (ATACseq) and expression data (RNAseq) revealed a core desiccation response across these two grasses. This includes many genes with binding sites for the core seed development transcription factor ABI5, supporting the long‐standing hypothesis that vegetative desiccation tolerance evolved from rewiring seed pathways. *Oropetium thomaeum* has a unique set of desiccation induced genes and regulatory elements associated with photoprotection, pigment biosynthesis, and response to high light, reflecting its adaptation of protecting the photosynthetic apparatus under desiccation (homoiochlorophyly). By contrast, *Eragrostis nindensis* has unique accessible and expressed genes related to chlorophyll catabolism, scavenging of amino acids, and hypoxia, highlighting its poikilochlorophyllous adaptations of dismantling the photosynthetic apparatus and degrading chlorophyll under desiccation. Together, our results highlight the complex regulatory and expression dynamics underlying desiccation tolerance in grasses.

## 1 INTRODUCTION

Water deficit was the most pervasive challenge faced by charophyte green algae during the colonization of land, and it has been a continual force shaping the evolution and diversification of plants. Plants have evolved a versatile arsenal of strategies to avoid or overcome water limitations. At the extreme, a small group of plants can survive prolonged desiccation for months to years until the return of water. Desiccation tolerance, or the ability to survive atmospheric drying, has been investigated for more than a century (Bewley, 1979; Bristol, 1919). Anhydrobiosis, or “life without water,” causes cells to enter a glassy or solid state and puts tremendous stress on all of the macromolecules and organelles. Polysomes are lost during drying, effectively halting protein synthesis, but they are quickly restored during rewetting (Bewley, 1979). Mitochondria and chloroplasts of most species swell and become ill defined during desiccation, and only species able to restore these organelles are associated with recovery from dry conditions (Wellburn & Wellburn, 1976; Sherwin & Farrant, 1998). Biochemical, physiological, and molecular mechanisms underlying desiccation tolerance have been thoroughly described, but comparatively little is known about the regulatory dynamics controlling this trait and what distinguishes desiccation tolerance from more typical drought responses (Gechev et al., 2021).

Desiccation tolerance requires tight coordination of numerous cellular processes and pathways to protect plants from the effects of water deprivation and photooxidative damage under excess light. Various osmoprotectants, heat shock proteins, reactive oxygen species scavengers, changes in membrane lipid composition, and late embryogenesis abundant (LEA) proteins have well‐documented roles in desiccation tolerance (Hoekstra et al., 2001). Along with these conserved responses, desiccation tolerant plants utilize two distinct strategies to mitigate photo‐oxidative damage. Homoiochlorophyllous species retain and protect their chlorophyll and thylakoids during desiccation, whereas poikilochlorophyllous species break down and rebuild chlorophyll, thylakoids, and components of the photosynthetic apparatus under anhydrobiosis using specialized membrane free plastids (desiccoplasts) (Tuba et al., 1994). These divergent strategies are associated with distinct rehydration and photosynthetic kinetics and have utility in different environments.

Vegetative desiccation tolerance is thought to have evolved through rewiring preexisting pathways, perhaps convergently across independent lineages. Desiccation tolerance mechanisms are broadly conserved between vegetative tissues and seeds, prompting the long‐standing hypothesis that desiccation tolerance evolved through rewiring existing seed pathways (Costa et al., 2017; Illing et al., 2005; VanBuren, 2017; VanBuren, Man Wai, et al., 2018). However, recent comparative experiments suggest this connection is more nuanced, as seed pathways also play an important role in typical drought responses of desiccation sensitive species (Pardo et al., 2020). Water deficit responses are broadly regulated by the phytohormone abscisic acid (ABA), and the ABA regulon has a major role in desiccation tolerance (Gaff & Oliver, 2013; Manfre et al., 2009; Shinozaki and Yamaguchi‐Shinozaki & Yamaguchi‐Shinozaki, 2007) and drought responsive pathways (Daszkowska‐Golec, 2016). Several transcription factor families including dehydration‐responsive element‐binding factor (DREB), basic leucine zipper (bZIP), and NAM‐ATAF‐CUC2 (NAC) are regulated by ABA and play integral roles in drought and desiccation responses (Nakashima et al., 2014; Takasaki et al., 2015; Wang et al., 2019; Yoshida et al., 2015). The unique and overlapping pathways between drought and desiccation and the regulatory machinery underlying these stress responses remain poorly understood.

The regulation of gene activity based on proximity to *cis*‐sequences or entire regions has been a long standing interest of the biological community (Baker, 1968) and has been the source of significant advancement in our understanding of eukaryotic gene regulation (Eissenberg, 1989). High quality analysis of plant chromatin structure and accessibility has been under investigation for several decades (Bowler et al., 2004). Recently, the use of assay for transposase accessible chromatin sequencing (ATAC‐seq) has become a powerful tool to reveal binding sites of transcription factors and correlates well with changes in gene expression in various tissues and cell types (Lu et al., 2017). ATAC‐seq has even been used to draw correlations between species to identify evolutionarily conserved chromatin regions (Lu et al., 2019). Chromatin dynamics have been profiled under several abiotic stresses including cold, heat, and flooding (Han et al., 2020; Liang et al., 2021; Reynoso et al., 2019; Wang et al., 2021), but little is known about chromatin changes during desiccation. One small‐scale investigation identified a desiccation induced promoter, but this study did not identify underlying cis‐regularoty elements or provide a (Smith‐Espinoza et al., 2007) clear picture of how chromatin is affected by the loss of water or the link between chromatin changes and gene expression.

Here, we surveyed changes in chromatin architecture, gene expression, and regulatory dynamics under desiccation in two related resurrection grasses with contrasting photoprotective strategies. We collected parallel datasets from the homoiochlorophyllous (chlorophyll retaining) model resurrection grass *Oropetium thomaeum* and the poikilochlorophyllous (chlorophyll degrading) grass *Eragrostis nindensis*. These species are found in the Chloridoideae subfamily of grasses, a group of diverse, stress tolerant species that includes the important underutilized crops teff (*Eragrostis tef*) and finger millet (*Eleusine coracana*). *O. thomaeum* also has the smallest diploid genome among grasses (~240 Mbp) (VanBuren, Wai, et al., 2018), and the compact monoploid genomes within this subfamily enable association between accessible chromatin regions (ACRs) and nearby genes. Previous studies in *O. thomaeum* showed an induction of pathways and gene families related to seed development such as ELIPs and LEAs during desiccation (VanBuren et al., 2017). Here, we sought to identify regulatory elements that enabled these unique pathways compared with typical drought responses. With the use of this comparative system, we show that significant shifts in chromatin architecture are associated with desiccation and photoprotective responses in resurrection grasses. We identified a core set of genes, accesable chromatin peaks, and *cis*‐regulatory elements that underly conserved desiccation tolernace responses across these two grasses. We also found distinct regulatory dynamics that are associated with the different strategies of retaining or degrading the photosynthetic apparatus under anhydrobiosis.

## 2 RESULTS

### 2.1 Dynamic patterns of chromatin accessibility under desiccation

**FIGURE 1:**
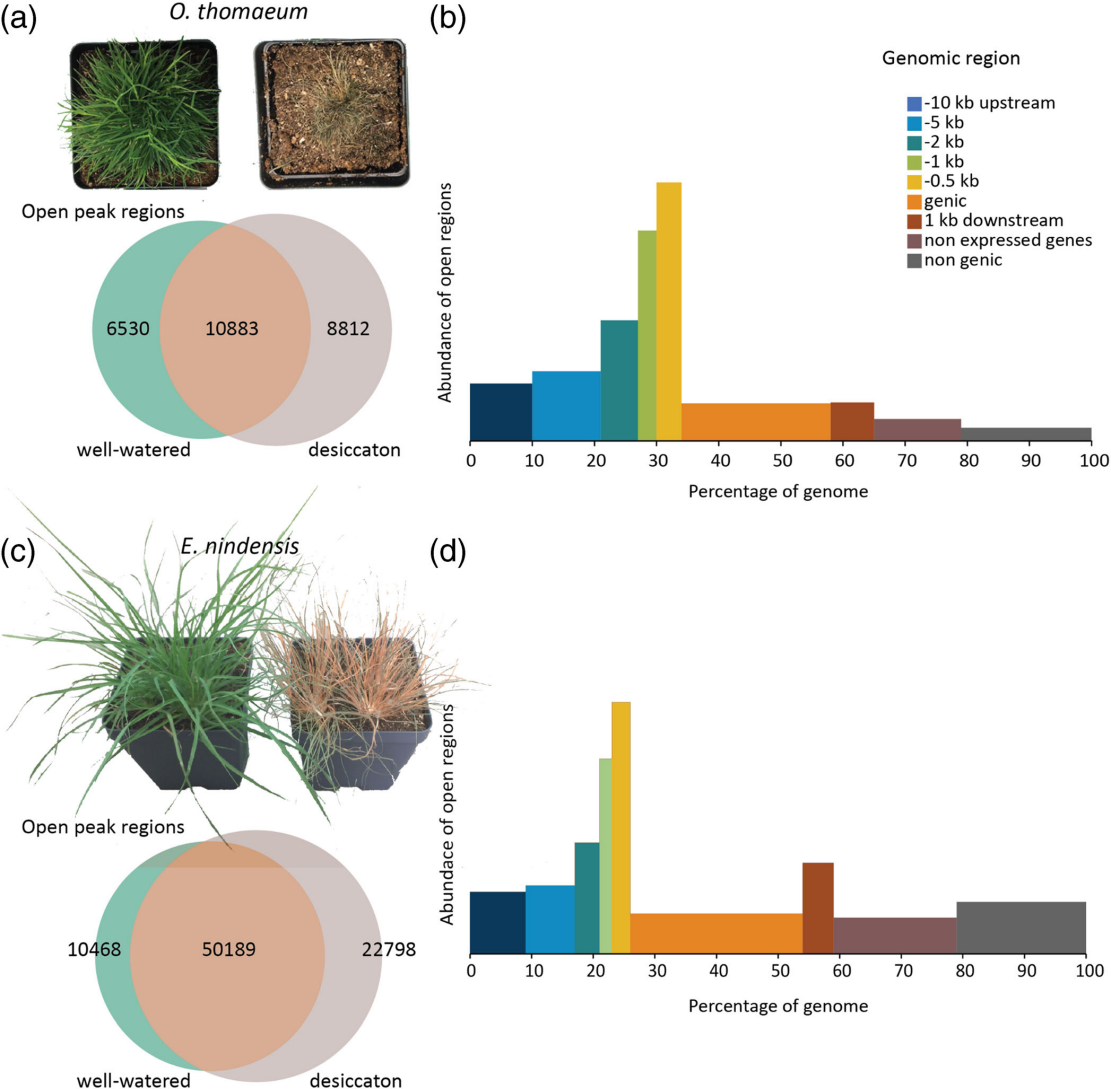
Chromatin architecture changes underlying desiccation responses in resurrection grasses. (a) Overlapping (orange) accessible chromatin regions (ACRs) between well‐watered (green) and desiccated (gray) *Oropetium thomaeum* plants. Representative well‐watered (left) and fully desiccated (right) *O. thomaeum* plants are shown. (b) Landscape of accessible chromatin regions across the *O. thomaeum* genome. The abundance of ACRs is shown on the Y axis and percentage of the genome corresponding to upstream, genic, downstream, intergenic, and genes with no detectable expression is shown on the X axis. (c) Overlapping (orange) ACRs between well‐watered (green) and desiccated (gray) *Eragrostis nindensis* plants. Representative well‐watered (left) and fully desiccated (right) *E. nindensis* plants are shown. (d) Landscape of ACRs across the *E. nindensis* genome

We surveyed the changes in chromatin architecture and gene expression underlying desiccation tolerance using a combination of ATAC‐seq and RNAseq in *O. thomaeum* and *E. nindensis*. Mature plants were slowly dried over a period of 10 days without watering to trigger desiccation, and samples for RNAseq and ATAC‐seq were collected in parallel from leaves of desiccated and well‐watered plants (Figure 1). RNAseq reads were quality trimmed, pseudo‐aligned to the *O. thomeaum* and *E. nindensis* gene models using Kallisto (Bray et al., 2016), and differentially expressed genes were identified using Sleuth (Pimentel et al., 2017). In *O. thomaeum*, 5,187 genes had higher expression under desiccation, and 5,354 genes had lower expression compared with well‐watered (*q* < 0.05). Similar timepoints in *E. nindensis* had 8,071 genes with higher expression under desiccation and 8,971 genes with lower expression compared with well‐watered. This represents roughly one third of the genes in the *O. thomaeum* genome and almost half of the genes with detectable expression. In *E. nindensis*, the differentially expressed genes represent about 15% of all genes and 30% of the genes with detectable expression. These expression dynamics highlight the complex transcriptional reprogramming required for the successful deployment of desiccation tolerance in both species. The *O. thomaeum* genes with the highest expression in desiccation include dehydrins (Ot_Chr8_n39154), early light induced proteins (ELIPs; Ot_Chr8_39125, Ot_Chr8_n38842), and other proteins with well‐characterized roles in desiccation and abiotic stress responses (Table S1). Genes with the highest differential expression in desiccated *E. nindensis* plants included two late embryogenesis‐related proteins (LEAs; En_0084954, En_0002557), an aldehyde dehydrogenase (En_0060658), and a glucosyl transferase (En_0017075; Table S2).

We utilized ATAC‐seq to profile chromatin openness and link expression dynamics with putative *cis*‐regulatory elements. Nuclei were isolated from flash frozen samples, and ~50,000 nuclei were used to construct ATACseq libraries for desiccated and well‐watered samples in each species. Naked DNA controls were also collected to mask regions showing TN5 insertional bias. Trimmed reads were aligned to the *O. thomeaum* V2 or *E. nindensis* V2 genomes using bowtie2 (Langmead & Salzberg, 2012), and peaks of accessible chromatin were called using Genrich (Gaspar, 2018). Genome browser views of the aligned ATAC‐seq reads show strong peaks of ACR compared to the background and naked DNA, as well as an association of peaks and genic regions for both species (Figures S1 and S2). Peaks are consistent across replicates and treatments, indicating the libraries are high quality (Figures S3 and S4). Differential peak calling with DiffBind (Stark et al., 2011) provided a Fraction of Reads in Peaks (FRiP) of between 0.26 and 0.47. The well‐watered samples all have lower FRiP (0.28, SD 0.02) than the desiccated samples (0.45, SD 0.03); however, fewer open regions were detected in desiccated samples with 17,257 versus 24,194 ACRs in *O. thomaeum*, and 55,514 versus 60,777 ACRs in *E. nindensis* (Tables S3 and S4).

In total, we identified 26,225 ACRs in *O. thomaeum* and 83,455 ACRs in *E. nindensis* occupying 6.4% and 4.9% of the genome, respectively (Figure 1a,c). Roughly 85% and 82% of all ACRs were near expressed genes in *O. thomaeum* and *E. nindensis*, respectively. For both species, the 0–500 bp bin upstream of the transcription start site (TSS) has an over‐representation of ACRs based on the relative proportion of the genome occupied by this range (Figure 1b,d). We also observed that ACR density decreases with distance from the TSS. The *O. thomaeum* and *E. nindensis* genomes are highly compact compared to other grassses (Pardo et al., 2020; VanBuren et al., 2015; VanBuren et al., 2020), and expressed genes and their flanking regions (i.e., 10 kb upstream to 1 kb downstream) cover 65.8% and 59.4% of their genomes, respectively. Not all ACRs were found near canonical genes. Of the open regions, 7.2% and 14.2% are near non‐expressed genes, and 7.5% and 21.5% of the regions were not near any genes.

### 2.2 Chromatin architecture is tightly linked to the regulation of desiccation responsive genes

**FIGURE 2:**
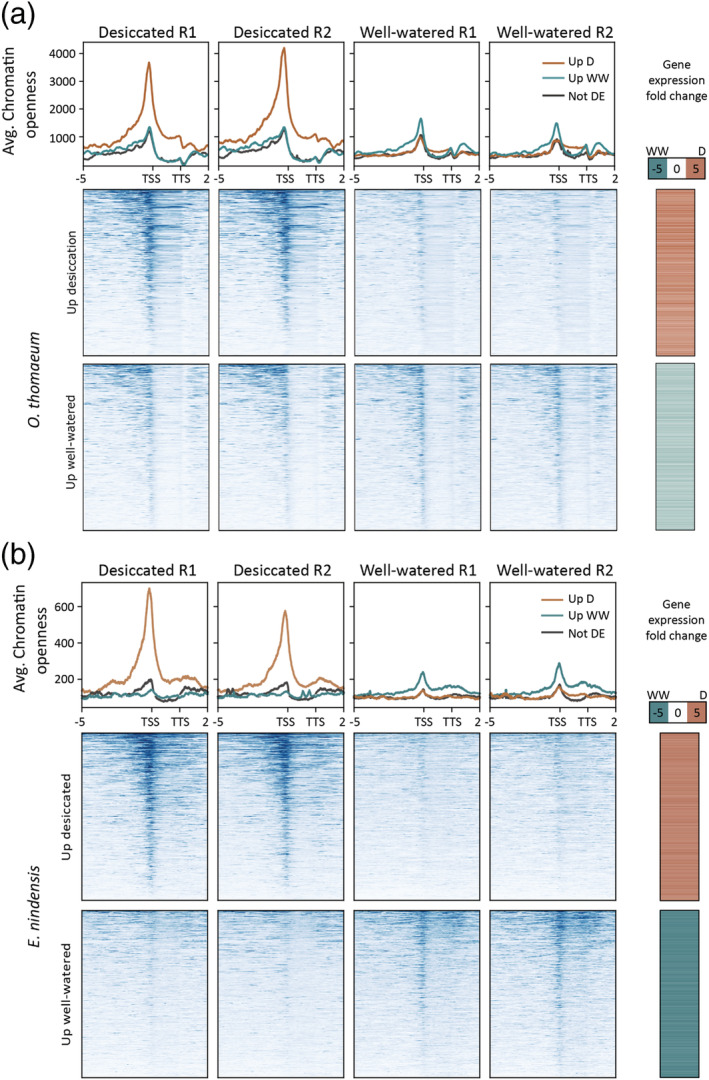
Chromatin architecture and gene expression associations under desiccation. The average chromatin openness (depth of reads) is plotted for genes upregulated under desiccation (orange) or upregulated under well‐watered (blue) for the two replicates in each sample for *Oropetium thomaeum* (a) and *Eragrostis nindensis* (b). The log2 fold change of expression is plotted on the right for each gene. Openness is plotted 5 kbp upstream to the TSS, on the gene, and the TTS to 2 kbp downstream. TSS, transcriptional start site; TTS, transcription termination site

Because chromatin architecture is believed to impact the expression level of nearby genes, we investigated the relationship between chromatin openness and gene expression using two different methods. Expressed genes typically have ACRs near the TSS (Figure 1b,d), and we used deepTools (Ramírez et al., 2016) to aggregate the genomic regions near differentially expressed genes. We found that ACRs near genes are significantly more open when the gene has higher expression (Figure 2). This correlation was strongest for genes with higher expression under desiccation in both *O. thomaeum* and *E. nindensis* (Figure 2a,b). Genes with upregulated expression in well‐watered tissues had a distinct peak in openness near the TSS, but this was less pronounced than desiccation induced genes. We then looked at differentially expressed genes that had a differentially accessible region in each bin and plotted the best‐fit line for each combination. The *R* ^2^ value for that line‐fit drops off inside the 5000 to 2000 bp region and is highest for the 0 to 500 bp region upstream of the TSS (Figure S5).

To quantify the impact of chromatin accessibility on expression dynamics, we split the differentially expressed genes into four categories depending on the direction of differential gene expression and chromatin openness. There are 18,344 expressed genes in *O. thomaeum*, and 15,617 (85.1%) have an ACR within 3,000 bps upstream (nearby). This proportion is similar for differentially expressed genes, where 9,030 of 10,541 (85.7%) have an ACR nearby. Genes with higher expression in one treatment were more likely to have a chromatin region that was more open nearby. Well‐watered and desiccated samples had 2,621 and 3,059 cases,d respectively, where a higher expressed gene was near a more open ACR. Whereas, there were only 1,159 and 2,077 cases of higher expression in desiccation with less open ACRs compared with well‐watered or vice versa (chi‐square [4, *N* = 26,225] = 10,414.16, *p* < 0.00001). In *E. nindnesis*, 36,932 of the 56,205 expressed genes (65.7%) and 12,651 of the 17,042 differentially expressed genes (74.2%) have an ACR nearby, respectively. The well‐watered and desiccated samples had 2,126 and 4,957 cases, respectively, where a higher expressed gene was near a more open ACR. The genes with higher expression in desiccation but a more open region in well‐watered included 572 cases, and there were 2,300 with the reverse (chi‐square [4, *N* = 83,455] = 12817.77, *p* < 0.00001).

**FIGURE 3:**
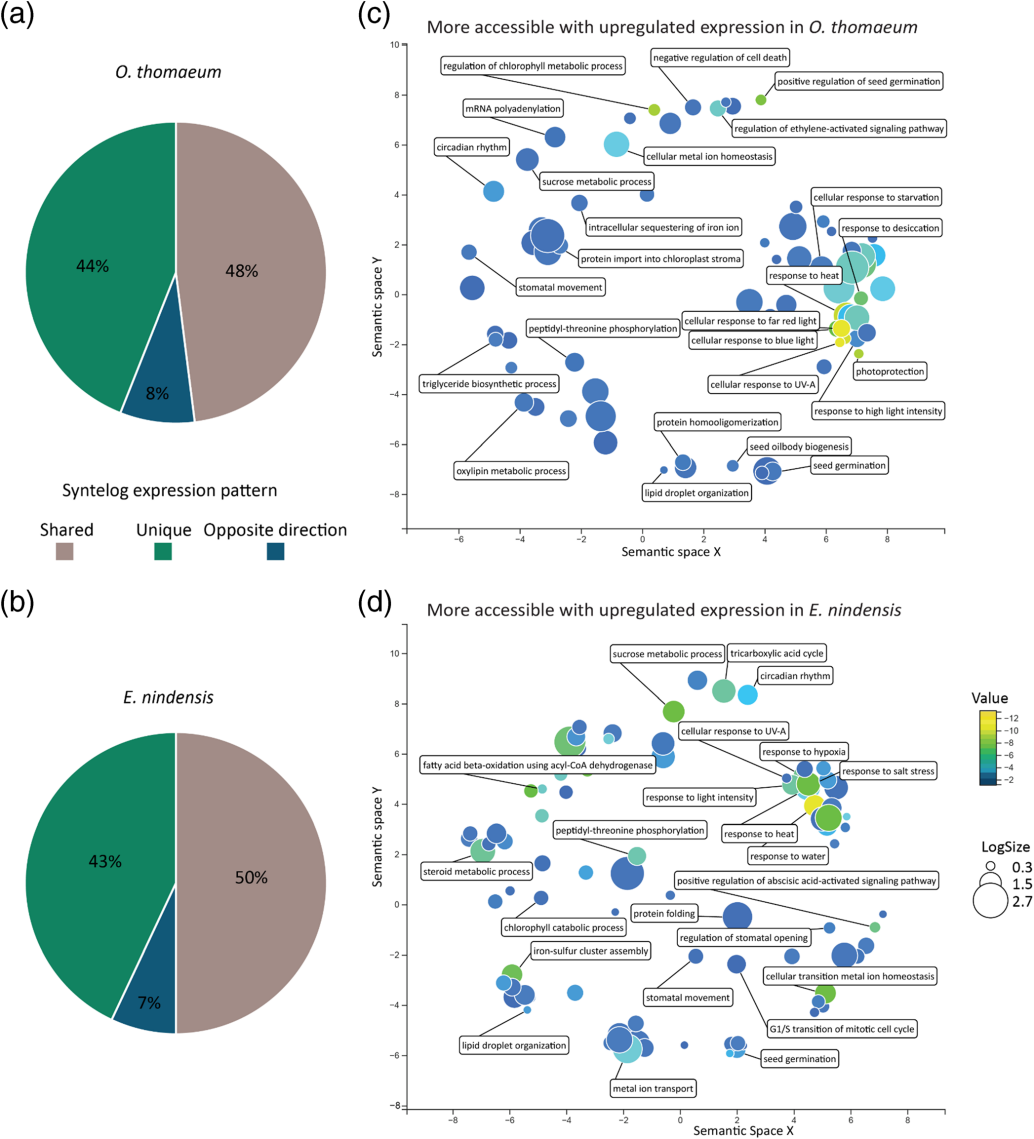
Contrasting enrichment patterns of desiccation‐associated genes in *Oropetium thomaeum* and *Eragrostis nindensis*. The proportion of syntelogs with similar, unique, or opposing expression patterns is plotted for *O. thomaeum* (a) and *E. nindensis* (b). Enriched gene ontology terms (GO terms) for genes that have more accessible chromatin and higher expression under desiccation are plotted for *O. thomaeum* (c) and *E. nindensis* (d). GO terms are transformed using multidimensional scaling to reduce dimensionality, and terms are grouped by semantic similarities. GO terms with previously characterized roles in desiccation and photoprotection responses are highlighted. The color of the circles represent significance and size represents the number of genes in that group.

To enable detailed comparisons of expression patterns and chromatin dynamics across species, we utilized a set of syntenic orthologs between the two grasses (Pardo et al., 2020). Roughly half of the differentially expressed syntenic orthologs in *E. nindensis* (49.8% or 6,685) and *O. thomaeum* (48.2% or 4,460) have conserved expression patterns under desiccation (Figure 3a,b; Tables S5 and S6). Comparatively few syntenic orthologs have the opposite expression pattern between species under desiccation with only 6.9% or 932 in *O. thomaeum* and 7.9% or 735 in *E. nindensis*. We searched for patterns of functional enrichment of syntenic orthologs with similar chromatin and expression dynamics that could indicate conserved underlying desiccation responses in grasses. Enriched gene ontology terms (GO terms) of upregulated syntelogs in both species include response to water deprivation and other abiotic stresses, fatty acid biosynthesis, golgi organization, proteasomes, and respiration (Figure 3c,d, Tables S7 and S8). GO terms associated with syntelogs that are similarly downregulated in desiccation are related to photosynthesis, core and secondary metabolism, cell wall processes, and hormone metabolism (Table S8). Broadly, this suggests these two grasses utilize fundamentally similar mechanisms to survive desiccation.

### 2.3 *Cis*‐regulatory elements associated with desiccation tolerance

With the use of these datasets of ACRs and gene expression, we identified putative *cis*‐regulatory elements that are involved in the regulation of desiccation responses. We extracted sequences within differential ACRs near expressed genes in *O. thomaeum* and *E. nindensis* and searched for enriched motifs within each set. We utilized the Motif Discovery and Enrichment Analysis (XSTREME) algorithm from Multiple Em for Motif Elicitation (MEME) to identify motifs and compare with motif sequences that have been previously investigated (Bailey et al., 2009; Grant and Bailey & Grant, 2021). De novo motifs were functionally annotated based on homology against the Arabidopsis transcription factor motif database using TOMTOM (Bailey, 2020; Bailey et al., 2009; Gupta et al., 2007; O'Malley et al., 2016).

**FIGURE 4:**
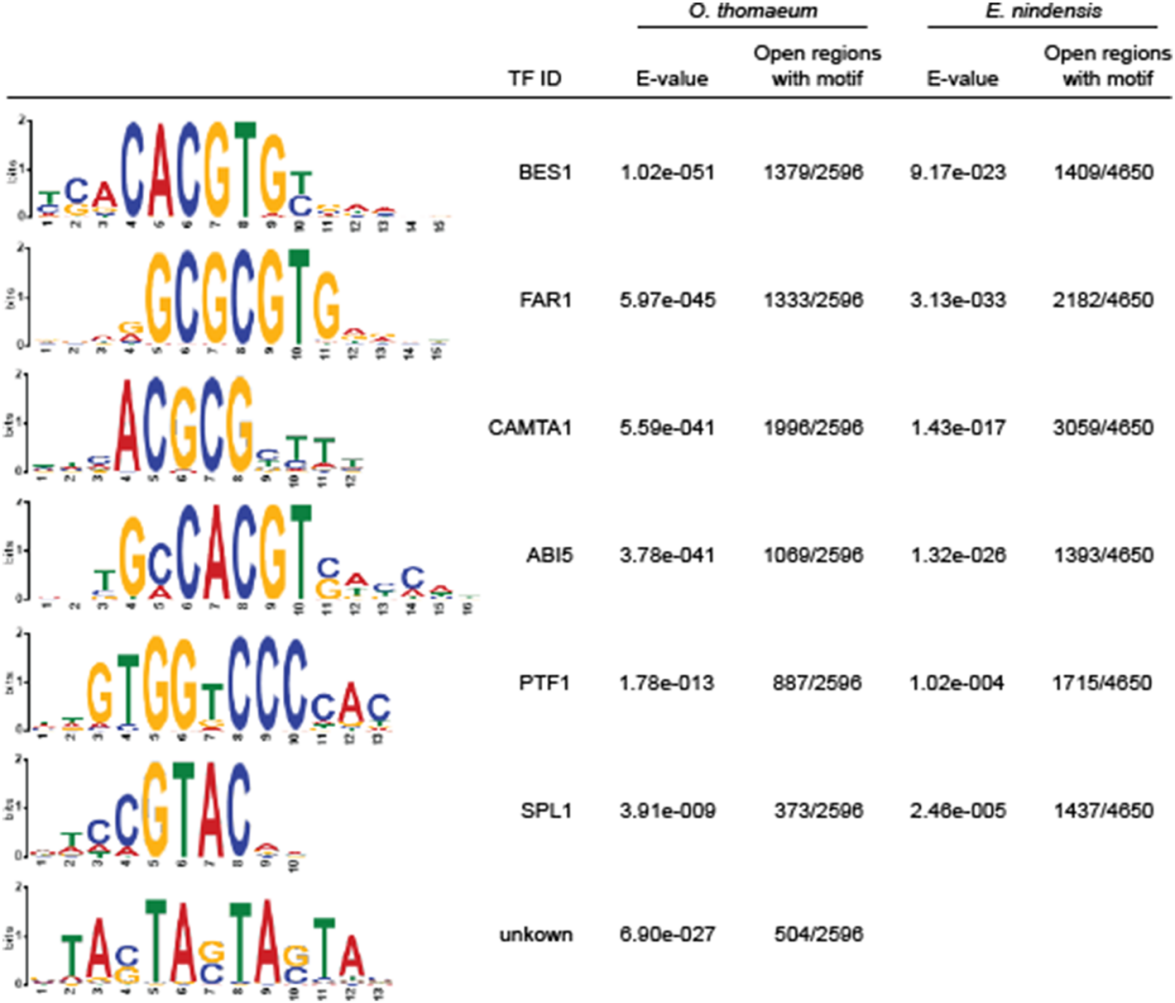
Enriched regulatory motifs associated with desiccation responsive genes. Enriched *cis*‐regulatory element motifs in accessible chromatin regions (ACRs) of genes upregulated under desiccation in both grasses are shown. The last logo (TF ID: unknown) was obtained from a de novo search for motifs. The motif logo, most closely associated transcription factor, statistical significance (*E* value), and number of open regions with each motif are shown for *Oropetium thomaeum* (left) and *Eragrostis nindensis* (right).

We identified 34 and 31 enriched (*E* value ≤ 5e‐02) *cis‐*element motifs from regions that were more open and near upregulated genes under desiccation in *O. thomaeum* and *E. nindensis*, respectively (Figure 4). More open ACRs near genes upregulated under well‐watered conditions have 21 and 20 enriched motifs in *O. thomaeum* and *E. nindensis*, respectively. Enriched motifs under desiccation in both grasses are similar (Figure 4) and resemble motifs for transcription factors associated with response to water stress (CAMTA1), heat stress (SPL1), seedling development (AREB3, ABI5), and ultraviolet (UV) protection/light signaling (BES1, PTF1, FAR1) (Kim et al., 2002) (Figure 4). STREAM identified 15 novel motifs from the *O. thomaeum* desiccation samples including a highly enriched motif with no homology in Arabidopsis, mouse, or fruitfly datasets (TA(G/C)TA(G/C)TA; 504 of 2596 sites; *E* value of 6.90e‐27) (Bailey, 2020). This motif may play a central role in anhydrobiosis related processes in grasses.

### 2.4 Regulatory dynamics distinguishing photoprotective strategies under desiccation

*O. thomaeum* and *E. nindensis* utilize different strategies to mitigate photooxidative damage under anhydrobiosis. *O. thomaeum* retains and protects, whereas *E. nindensis* degrades and resynthesizes chlorophyll, thylakoid membranes, and components of the photosynthetic apparatus during desiccation and rehydration cycles (Bartels & Mattar, 2002). We searched for unique ACRs, expression dynamics, and putative *cis‐*regulatory elements that distinguish the distinct desiccation tolerance strategeis in these two grasses. The sets of syntenic orthologs and ACRs between *O. thomaeum* and *E. nindensis* were used for comparative analyses.

Syntenic orthologous or species‐specific genes that are uniquely upregulated in *O. thomaeum* compared with *E. nindensis* have functions related to cellular response to heat and high light, photoprotection, protein localization to chloroplast, regulation of seed germination, and regulation of cell cycle (Table S8). Upregulated genes with more open ACR in *O. thomaeum* have similar enrichment patterns including regulation of chlorophyll metabolic process, positive regulation of seed germination, cellular response to high light intensity, and cellular response to blue light (Table S9). Uniquely downregulated genes under desiccation in *O. thomaeum* were enriched in GO terms associated with detection of bacteria and icosanoid metabolic process (Table S8). Genes that are uniquely upregulated in *E. nindensis* under desiccation include enriched GO terms related to tricarboxylic acid cycle, isocitrate metabolic process, response to hypoxia, and leucine metabolic process. Uniquely downregulated genes under desiccation in *E. nindensis* are enriched in GO terms associated with photosystem II assembly (Table S8).

**FIGURE 5:**
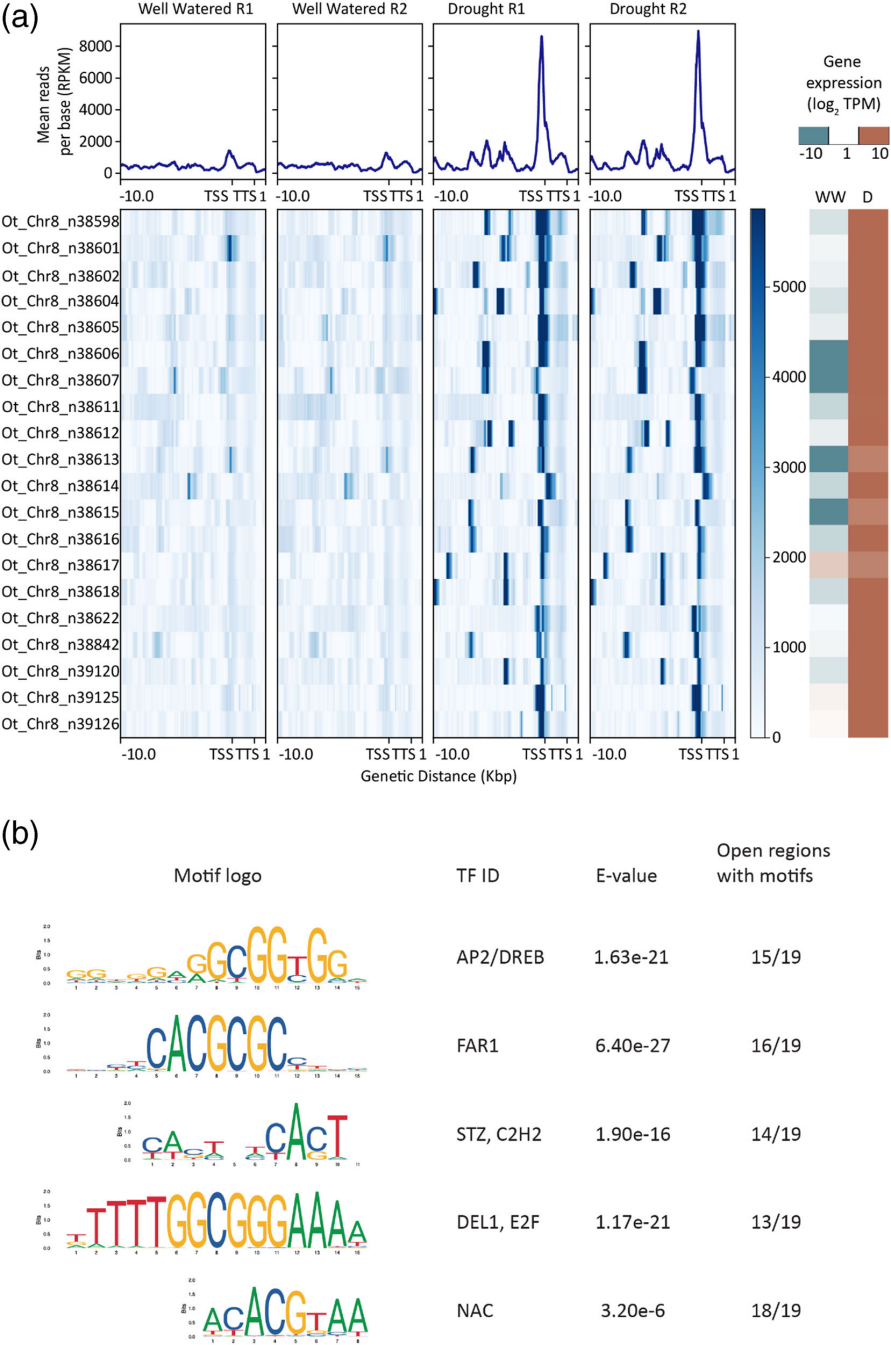
Regulatory dynamics of early light induced proteins (ELIPs) under desiccation. (a) Chromatin architecture and expression dynamics of ELIPs in well‐watered and desiccated *Oropetium thomaeum* samples. The mean mapped read depth of ATACseq reads (in RPKM) is plotted for 10 kb upstream to 1 kb downstream regions for each of the ELIPs in the *O. thomaeum* genome. Log2 transformed RNA expression (in TPM) for each ELIP is shown on the right under well‐watered and desiccated conditions. (b) Enriched putative *cis*‐element motifs in accessible chromatin regions (ACRs) upstream of ELIP TSSs in *O. thomaeum*. The motif logo, associated transcription factor (TF ID), *E* value, and number of open regions with each motif are shown.

The evolution of desiccation tolerance is associated with massive duplication of ELIPs, which play a central role in photoprotection under anhydrobiosis (VanBuren et al., 2019). All sequenced resurrection plants contain large tandem arrays of ELIPs including *O. thomaeum* (20 ELIPs) and *E. nindensis* (27), but chlorophyll retaining species typically have more ELIPs when accounting for ploidy (VanBuren et al., 2019). The 20 ELIPs in *O. thomaeum* are among the most highly expressed genes under desiccation, with on average >20‐fold higher expression compared to well‐watered (Figure 5a). The ELIPs have no significant ACR peaks under well‐watered conditions but have massive peaks of ACRs upstream of the TSS under desiccation (Figure S6). All but four of the ELIPs in *O. thomaeum* are found in a single tandem array with high sequence homology. Interestingly, some genes within this array have unique peaks in different positions upstream of the TSS, likely corresponding to changes in non‐coding sequences during their duplication. In the conserved peak upstream of each ELIP TSS, we found a highly enriched *cis‐*regulatroy motif for the central ABA responsive drought transcription factor AP2/DREB (Figure 5b). This motif is notably absent from the two Arabidopsis ELIPs and may be related to the evolution of desiccation tolerance. Several other enriched *cis*‐regulatory motifs were identified in a subset of peaks near ELIPs related to light responses (FAR1), ABA signaling (STZ, C2H2) and (ATAF1, NAC), and cell cycle regulation (DEL1, E2F) (Figure 5b).

Of the 27 ELIPs in *E. nindensis*, two ELIPs are not expressed, 14 are expressed similarly in well‐watered and desiccated tissues, seven are down regulated under desiccation, and four are upregulated under desiccation (Figure S7). Two of the 23 ACRs near *E. nindensis* ELIPs were significantly more open under desiccation and near genes with higher expression in desiccation, but the remainder showed no changes. These two differentially open regions contain sequences similar to the AP2/DREB motifs found near the *O. thomaeum* ELIPs. However, a search for enriched motifs among open regions near all *E. nindensis* ELIPs did not result in an overlap with motifs found in *O. thomaeum* ELIP open regions. Together, this highlights the important role that ELIPs play in chlorophyll retaining species and a possible regulatory neofunctionalization during the evolution of desiccation tolerance.

## 3 DISCUSSION

Desiccation tolerance has evolved recurrently across the tree of life as a common adaptation to survive anhydrobiosis in water‐limited environments. Tolerance is not a static or monolithic trait, and it has been repeatedly gained, lost, or modified across plant evolution. Here, we surveyed changes in chromatin architecture and gene expression in two related resurrection grasses that utilize different photoprotective strategies under anhydrobiosis. *O. thomaeum* and *E. nindensis* have conserved gene content and relatively small monoploid genome sizes (250 and 500 Mb, respectively), enabling detailed comparative genomic analyses and associations between putative *cis*‐regulatory elements and gene function. ACRs were found in largely genic regions, and desiccation induced shifts in chromatin accessibility for 10.7 Mb (4.5%) and 24.2 Mb (2.5%) of the *O. thomaeum* and *E. nindensi*s genomes, respectively. These shifts in accessibility are highly correlated with gene expression dynamics, and these high‐quality datasets allowed us to explore the unique biophysical constraints of anhydrobiosis and the regulatory evolution of this complex adaptation.

### 3.1 Chromatin architecture and anhydrobiosis

Chromatin dynamics have not been described for desiccated vegetative tissues, but chromatin is highly condensed and compacted in desiccated seeds (van Zanten et al., 2011). Differential chromatin accessibility is established in developing seeds, and gene level modifications enable rapid transcription for germination related processes upon hydration (Fransz & de Jong, 2011). We observed a high signal‐to‐noise ratio (FRiP) of the ACRs in desiccated leaf tissue stemming from a significantly lower background level of reads compared with well‐watered leaf tissue. This enrichment resulted in a higher abundance of reads in open peaks, potentially indicating a major difference in chromatin state in the nucleus of desiccated cells.

We hypothesize several factors that may contribute to the low background signal and robust peaks observed under desiccation. The first is that all nuclei in the desiccated plants are more condensed, and this compaction and associated factors prevent Tn5 accessibility in background regions. The more parsimonious explanation is that desiccated cells and nuclei are highly uniform with fewer cells actively undergoing transient processes that open chromatin, such as transcription or replication. Under desiccation, all cell types must arrest their normal developmental or metabolic functions and enact a series of highly coordinated responses to successfully prepare for anhydrobiosis and subsequent rehydration. In turn, this would “synchronize” cells and produce the observed tight coordination between expression dynamics and chromatin architecture that is typically only found in single cell data (Farmer et al., 2021). Drying of this magnitude is experienced by all cells, and risky processes such as growth and photosynthesis during the stresses of anhydrobiosis would be detrimental and preferentially avoided. Thus, a clear, tightly regulated signal from all cells in the desiccated samples is achieved. This is supported by the massive transcriptional reprogramming we observed, and the clear enrichment of GO terms related to photoprotective and anhydrobiosis processes with comparatively few background pathways. This pattern of clearer peaks and more accessible chromatin under desiccation contrasts what has been observed under drought and salinity (Raxwal et al., 2020) but is similar to cold stress (Zeng et al., 2019).

### 3.2 Evolutionary dynamics of photoprotective strategies under desiccation

*O. thomaeum* and *E. nindensis* are found within the Chloridoideae subfamily of grasses, a group of stress tolerant C4 species with superior drought, heat, and salinity tolerance (Marcum, 1999; Peterson et al., 2001). Evolutionary access to existing resilience traits within this subfamily likely enabled the independent evolution of desiccation tolerance in *O. thomaeum* and *E. nindensis* (Pardo & VanBuren, 2021). With the use of detailed comparative genomics approaches with syntenic orthologs, we identified a core set of genes and *cis*‐regulatory regions that are induced during desiccation in *O. thomaeum* and *E. nindensis*. Integration of ATAC‐seq and RNAseq data allowed us to filter out spurious associations, and we identified numerous enriched genes with previously described roles in desiccation. Among the stress responsive *cis*‐elements, we identified numerous binding motifs for the seed desiccation transcription factor ABI5, supporting the hypothesis that vegetative desiccation tolerance evolved from rewiring existing seed pathways (Oliver et al., 2000; Oliver et al., 2005). This hypothesis is contentious, and previous work in the monocot *Xerophyta humilis* failed to find evidence linking desiccation processes to the canonical seed development transcription factor network of LEC1, ABI3, ABI5, and others (Lyall et al., 2020). Our results provide a direct link between expression dynamics and regions of accessible chromatin with seed related regulatory motifs, but this association needs to be further tested using transcription factor binding data.

Species‐specific changes in chromatin architecture and expression dynamics during desiccation reflect the distinct strategies that *E. nindensis* and *O. thomaeum* utilize to mitigate photooxidative damage. We identified a cluster of desiccation associated GO terms related to chlorophyll processes, UV‐A responses, photoprotection, and pigment metabolism that was unique to *O. thomaeum*. These orchestrated responses in *O. thomaeum* reflect homoiochlorophyly, or the strategy to protect chlorophyll and photosynthesis related macromolecules during desiccation. Consistent with this, *O. thomaeum* has more ELIPs than *E. nindensis* (when accounting for ploidy), and all of the ELIPs have massive shifts in chromatin accessibility and high expression under desiccation. By comparison, only two ELIPs in *E. nindensis* have associated ACRs, and few are differentially expressed under desiccation. Instead, ELIPs in *E. nindensis* have high expression during rehydration and likely function in protecting leaves as they resynthesize and repair their photosynthetic apparatus (Pardo et al., 2020).

We identified a *cis*‐regulatory motif associated with the drought transcripton factor DREB upstream of each ELIP in *O. thomaeum*. This motif is missing from the Arabidopsis ELIP orthologs and may represent a regulatory neofunctionalization to induce ELIP accumulation under desiccation. Nearly all plants have retained desiccation tolerance in their seeds and/or pollen, so the genes and regulatory elements needed to protect cells from anhydrobiosis are already in place. Tolerance may have evolved in resurrection plants through simply shifting the timing and cell specificity of existing desiccation pathways through *cis*‐regulatory elements, and our findings in ELIPs may be evidence of this process in action.

The GO terms we found imply desiccated plants were under stress responding to desiccation and a general downregulation of photosynthesis and carbon fixation in desiccated plants compared with the well‐watered. Both species also have an increase in genes associated with production of vitamin B6 under desiccated conditions. While vitamin B6 is associated with scavenging reactive oxygen, it is unclear if B6 levels are higher in desiccated tissues or what function these compounds are performing. Desiccated *O. thomaeum* also have enriched GO terms related to response to high light intensity, and a preservation of light‐harvesting, whereas in *E. nindensis*. there were enriched GO terms pertaining to catabolism of branched‐chain amino‐acids, endocytic recycling, and autophagy. Additionally, while both species had a down regulation of photosystem I, *E. nindensis* also had a downregulation of photosystem II. These differences highlight the nuanced evolution of desiccation tolerance, where a combination of deeply conserved pathways and species specific adaptations underlie this complex trait.

## 4 METHODS

### 4.1 Plants, samples, and growth conditions

*O. thomaeum* and *E. nindensis* plants were grown from seed for ~60 days for each experiment in a growth chamber with 28°C (day)/22°C (night) and 12 h light/12 h dark cycle. Desiccated samples were collected from plants where water was withheld for 10 days, causing the leaf relative water content to drop below 10%. Each replicate consisted of 3 mature plants grown in the same pot, and two replicates were collected for well‐watered and desiccated time points. Two grams of leaf tissue was collected from each plant and frozen in liquid nitrogen immediately. Tissues were ground into coarse powder and aliquoted into multiple 1.5 ml tubes with ~100 mg per tube. The samples were then subjected to nuclei isolation for ATAC‐seq or RNA extraction for RNAseq.

### 4.2 RNAseq library construction

RNA was extracted using Zymo research Direct‐ziol RNA miniprep kit according to the manufacturer's protocol with on‐column DNase digestion. RNA yield was quantified using a Qubit RNA BR kit, and RNA integrity was quantified by gel electrophoresis. One microgram of RNA was used to construct the RNAseq libraries using Illumina TruSeq stranded mRNA kit, according to the manufacturer's protocol. The multiplexed libraries were sequenced at Michigan State University RTSF Genomics Core with HiSeq4000 150 bp paired end mode for *O. thomaeum* and 100 bp paired end mode for *E. nindensis*.

### 4.3 ATAC‐seq library construction

Crude nuclei extracts were prepared using the protocol reported in Lu et al. (Lu et al., 2017). Briefly, approximately 0.5 g of liquid nitrogen ground tissue was resuspended in 10 ml of isolation buffer (15 mM Tris pH 7.5, 2 mM EDTA, 80 mM KCL, 20 mM NaCl, 15 mM 2‐Mercaptoethanol, 0.15% Triton‐X 100, and 0.5 mM Spermine) in a 50 ml falcon tube. Tissue was very gently agitated on ice for 15 min and filtered through 4 layers of miracloth followed by filtration through a 20 μm filter. The resulting filtrate was carefully pipetted onto an equal volume of Density Gradient Centrifugation Buffer (1.7 M Sucrose, 10 mM Tris–HCl pH 8.0. 2 mM MgCl2, 5 mM 2‐Mercaptoethanol, 1 mM EDTA, 0.15% Triton‐X 100) to create two phases. The sample was centrifuged at 2,500*g* for 30 min at 4°C, and the thin film of nuclei at the bottom of the tube were retained and resuspended in 600 μl of isolation buffer. Nuclei were stained with 4,6‐diamidino‐2‐phenylindole (DAPI) and counted using a Nikon Eclipse Ni‐Upright microscope with a 40× differential interference contrast objective lens and 50,000 nuclei were subjected to 2 μl of Tn5 enzyme digestion for 30 min at 37°C. DNAseq libraries were constructed from the resulting digested nucleosomes using the Illumina Nextera XT kit according to the manufacturer's protocol. The multiplexed libraries were sequenced at Michigan State University RTSF Genomics Core using an Illumina HiSeq4000 under 150 bp paired end mode.

Naked DNA controls were prepared for *O. thomaeum* using tissue from the well‐watered and desiccated samples. Approximately 50 ng of isolated genomic DNA was used for Tn5 tagmentation library construction as described above, and the multiplexed libraries were sequenced at Michigan State University RTSF Genomics Core with HiSeq4000 150 bp paired end mode.

### 4.4 RNAseq analysis

Paired‐end raw reads were trimmed using Trimmomatic (v0.33) (Bolger et al., 2014) to remove adapters and low‐quality bases. Reads were pseudo‐aligned to the *O. thomaeum* v2.1 or *E. nindensis* v2.1 transcriptomes using Kallisto (0.46.0) (Bray et al., 2016) with default parameters. Differential expressed genes (*q* value < 0.05) between well‐watered and drought conditions were identified using Sleuth (Pimentel et al., 2017).

### 4.5 ATAC‐seq analysis

Paired‐end raw reads were trimmed using Trim_galore (v0.6.6) to remove Nextera adapters and low‐quality bases (Krueger, 2018). Filtered reads were then aligned to the *O. thomaeum* v2.1, or *E. nindensis* v2.1 genomes using Bowtie2 (v2.4.2) with dovetailing reads enabled and very‐sensitive‐local enabled. Mapped reads were visualized using the Integrative Genomics Viewer (v2.12.3) (Robinson et al., 2011) to determine uniformity of coverage. A blackout list of genomic regions with abnormal read coverage was generated by investigating genes near large regions with greater than 100× coverage relative to other parts of the chromosome. Regions with both exceptionally high coverage and genes associated with the chloroplast were considered chloroplast contamination. A total of 25 regions consisting of about 1.5 Mbp of the ~236 Mbp *O. thomaeum* genome and 772 regions consisting of about 3.5 Mbp of the ~986 Mbp *E. nindensis* genome were added to the blackout lists. High mappability regions were identified in *O. thomaeum* by peak calling using Genrich with the naked DNA reads. These 2,562 high mappability regions consisting of ~1.6 Mbp were then used as a grey‐list for subsequent peak calling. Peaks associated with ACR were called using Genrich (v0.6.1) (Gaspar, 2018) with options specific for ATAC‐seq. Additionally, reads mapped from naked DNA were used as control for *O. thomaeum*. The resulting narrowPeak files and the read files (bam format) were used in the R library DiffBind (v3.4.11) (Ross‐Innes et al., 2012; Stark et al., 2011) to determine differentially open regions. DESeq2 (Love et al., 2014) was used to quantify differential ACRs, with normalization using the background library size.

### 4.6 Integrating ATACseq and RNAseq datasets

A genome‐wide assessment of ACR distribution was performed using deepTools (v3.4.3) (Ramírez et al., 2016). ACRs were classified into different groups based on their proximity to genes, and comparisons of peak openness and differential gene expression were performed. These correlations were used as input for gene ontology and cis‐element enrichment analyses.

We identified genes near ACRs using bedTools (v2.29.2) (Quinlan, 2014). ACRs were grouped into different categories based on their distance to genic elements including: 10,000 to 5,001, 5,000 to 2,001, 2,000 to 1,001, 1,000 to 501, 500 to 0 bp upstream (5′) of the TSS; overlapping with the gene; and 0 to 1,000 bp downstream (3′) of the transcription termination site (TTS). On the basis of the distribution and correlation of chromatin openness and gene expression patterns, we chose the range of 0–3,000 bp upstream of gene TSSs as the cutoff of associating ACRs with specific genes. The *O. thomaeum* and *E. nindensis* genomes are relatively compact compared to other grass species, and putative *cis*‐regulatory regions are in close proximity to genes.

Genes were grouped by their differential expression patterns and ACR, and GO term enrichment analyses were performed using the R library topGO (v2.46) (Alexa & Rahnenführer, 2009). Six different sets of genes from each species were used as inputs for GO term enrichment including up and downregulated genes under desiccation with no comparison with ACRs and the four categories of gene expression and ACR openness. This included (1) upregulated genes near more open ACRs under desiccation, (2) less open ACRs and downregulated expression, (3) less open ACRs and upregulated expression, or (4) more open ACRs and downregulated expression.

Motifs associated with ACRs within 3 Kbp upstream of differentially expressed genes were identified, and the Motif Discovery and Enrichment Analysis (XSTREME) algorithm from Multiple Em for Motif Elicitation (MEME) (v5.4.1) was used to identify enriched motifs in our data and associate these motifs with known transcription factor binding sites (Bailey and Grant & Bailey, 2021).

### 4.7 Comparative genomic analyses

Comparative genomic analyses were conducted between *O. thomaeum* and *E. nindensis* to identify shared and species‐specific sets of differentially expressed genes and ACRs dynamics. Syntenic orthologs were identified between the two closely related grasses using the python version of MCScan (https://github.com/tanghaibao/jcvi/wiki/MCscan-(Python-version)) (Wang et al., 2012). The chromosome scale *O. thomaeum* genome was used as an anchor, and the two sets of homeologous genes in the *E. nindensis* genome were mapped to the single corresponding syntenic ortholog in *O. thomaeum*. Genes in each species were then classified as syntenic or non‐syntenic, and these two designations were incorporated with differential expression analyses, ACR dynamics, and enriched motif analyses. Enriched GO terms and putative *cis*‐element motifs were identified for syntenic orthologs with conserved and species‐specific expression patterns as described above.

## CONFLICT OF INTEREST

The Authors did not report any conflict of interest.

## Supporting information


**Figure S1:** Genome Browser view of ATAC‐seq and RNA‐seq data near a region with differentially expressed genes in *Oropetium thomaeum*. ACR peaks, aligned bam files of ATACseq reads, differentially expressed genes, and the gene structure tracks are shown for a random region of chromosome 3.
**Figure S2:** Genome Browser view of ATAC‐seq and RNA‐seq data near a region with differentially expressed genes in *Eragrostis nindensis*. ACR peaks, aligned bam files of ATACseq reads, differentially expressed genes, and the gene structure tracks are shown for a random region of chromosome 3.
**Figure S3:** Scaled plot of accessible chromatin regions near genes in *O. thomaeum*. Scaled peaks are plotted for the 1 kb upstream, downstream, and genic regions.
**Figure S4:** Scaled plot of open regions and surrounding chromatin area *E. nindensis*. Scaled peaks are plotted for the 1 kb upstream, downstream, and genic regions.
**Figure S5:** Correlation between differential gene expression and nearby chromatin openness in (a) *O. thomaeum* and (b) *E. nindensis*. The differentially expressed genes in desiccation with differentially open chromatin within denoted regions are plotted. Genes in the upper right hand quadrant are more open with higher expression under desiccation. Genes in the lower left hand quadrant have lower expression and less chromatin openness under desiccation.
**Figure S6:** Genome Browser view of ATAC‐seq data near the ELIP tandem gene array in *Oropetium thomaeum*. Aligned bam files for the naked DNA and ATACseq reads and the gene structure tracks are shown.
**Figure S7:** Regulatory dynamics of ELIPs in *E. nindensis.* Chromatin architecture and expression dynamics of ELIPs in well‐watered and desiccated *E. nindensis* samples. The mean mapped read depth of ATACseq reads (in RPKM) is plotted for 10 kb upstream to 1 kb downstream regions for each of the ELIPs in the *O. thomaeum* genome. Log2 transformed RNA expression (in TPM) for each ELIP is shown on the right under well‐watered and desiccated conditions.

**Table S1:** Differential gene expression in *O. thomaeum*

**Table S2:** Differential gene expression in *E. nindensis*

**Table S3:** Differential chromatin openness in *O. thomaeum*

**Table S4:** Differential chromatin accessibility in *E. nindensis*

**Table S5:** Syntenic differential expression in *O. thomaeum* and *E. nindensis*

**Table S6:** Syntenic chromatin accessibility in *O. thomaeum* and *E. nindensis*

**Table S7:** Gene Ontology Heatmap

**Table S8:** Gene Ontology for different sets of syntenic genes

**Table S9:** Gene Ontology for different sets of genes and chromatin openness

## Data Availability

The raw RNAseq and ATACseq data are available from the National Center for Biotechnology Information (NCBI) Short Read Archive. Raw data for this project can be found under BioProject accession no. PRJNA807505.
