# Supplementary material for: Regulatory dynamics distinguishing desiccation tolerance strategies within resurrection grasses
Source: Plant Direct. 2022 Dec 13;6(12):e457. doi: 10.1002/pld3.457 (PMC9748243; doi:10.1002/pld3.457)
Supplement: Supplementary file 1 — Figure S1: Genome Browser view of ATAC‐seq and RNA‐seq data near a region with differentially expressed genes in Oropetium thomaeum. ACR peaks, aligned bam files of ATACseq reads, differentially expressed genes, and the gene structure tracks are shown for a random region of chromosome 3. Figure S2: Genome Browser view of ATAC‐seq and RNA‐seq data near a region with differentially expressed genes in Eragrostis nindensis. ACR peaks, aligned bam files of ATACseq reads, differentially expressed genes, and the gene structure tracks are shown for a random region of chromosome 3. Figure S3: Scaled plot of accessible chromatin regions near genes in O. thomaeum. Scaled peaks are plotted for the 1 kb upstream, downstream, and genic regions. Figure S4: Scaled plot of open regions and surrounding chromatin area E. nindensis. Scaled peaks are plotted for the 1 kb upstream, downstream, and genic regions. Figure S5: Correlation between differential gene expression and nearby chromatin openness in (a) O. thomaeum and (b) E. nindensis. The differentially expressed genes in desiccation with differentially open chromatin within denoted regions are plotted. Genes in the upper right hand quadrant are more open with higher expression under desiccation. Genes in the lower left hand quadrant have lower expression and less chromatin openness under desiccation. Figure S6: Genome Browser view of ATAC‐seq data near the ELIP tandem gene array in Oropetium thomaeum. Aligned bam files for the naked DNA and ATACseq reads and the gene structure tracks are shown. Figure S7: Regulatory dynamics of ELIPs in E. nindensis. Chromatin architecture and expression dynamics of ELIPs in well‐watered and desiccated E. nindensis samples. The mean mapped read depth of ATACseq reads (in RPKM) is plotted for 10 kb upstream to 1 kb downstream regions for each of the ELIPs in the O. thomaeum genome. Log2 transformed RNA expression (in TPM) for each ELIP is shown on the right under well‐watered and desiccated conditi [file PLD3-6-e457-s006.docx]

**Supplemental materials**


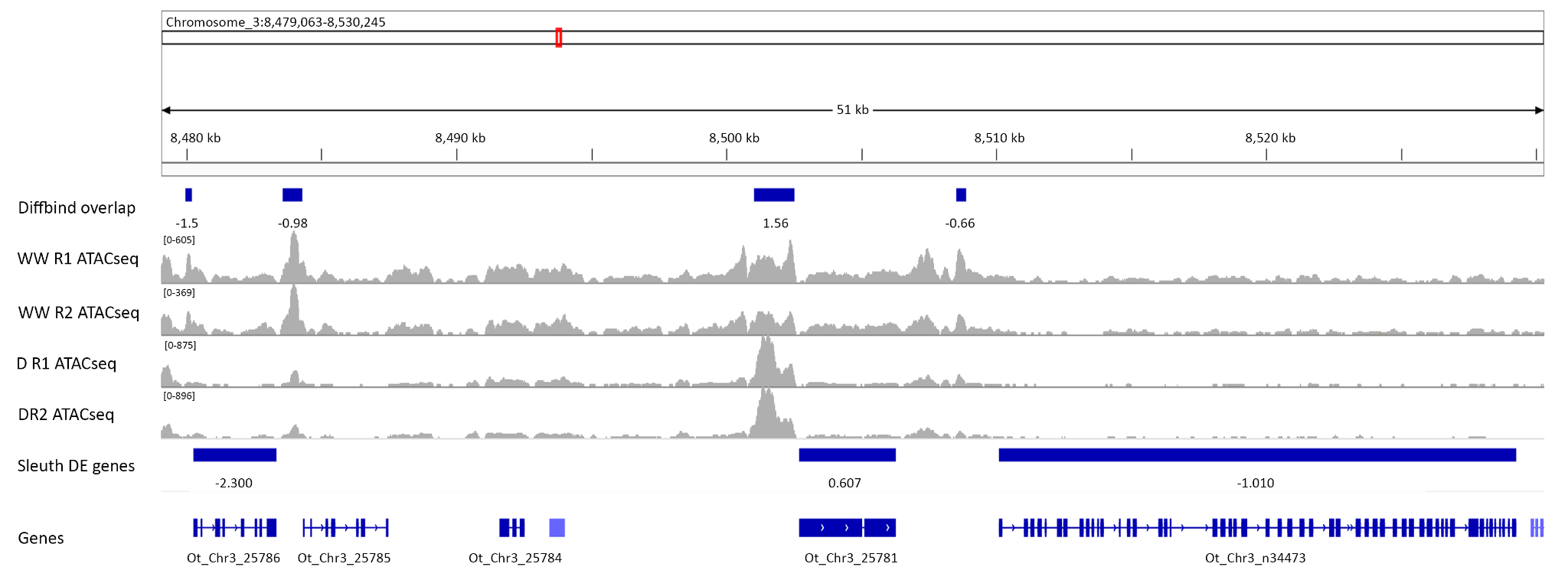


**Fig S1: Genome Browser view of ATAC-seq and RNA-seq data near a region with differentially expressed genes in *Oropetium thomaeum*.** ACR peaks**,** aligned bam files of ATACseq reads, differentially expressed genes, and the gene structure tracks are shown for a random region of chromosome 3.


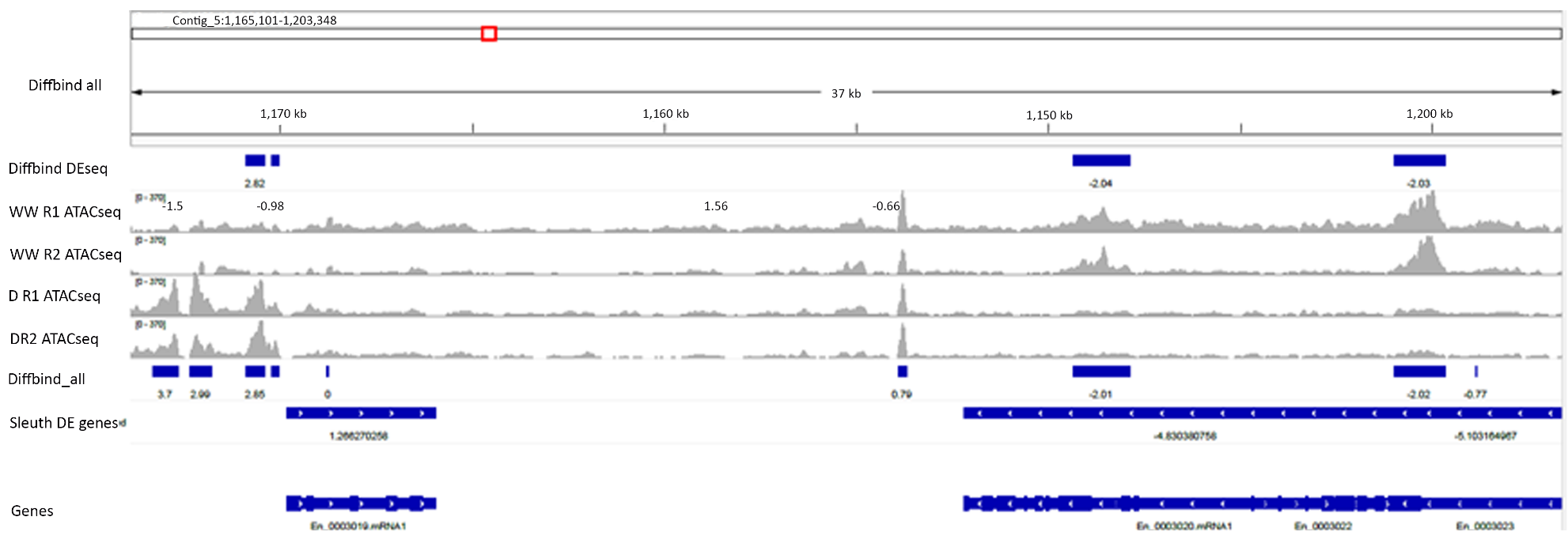


**Fig S2: Genome Browser view of ATAC-seq and RNA-seq data near a region with differentially expressed genes in *Eragrostis nindensis*.** ACR peaks**,** aligned bam files of ATACseq reads, differentially expressed genes, and the gene structure tracks are shown for a random region of chromosome 3.


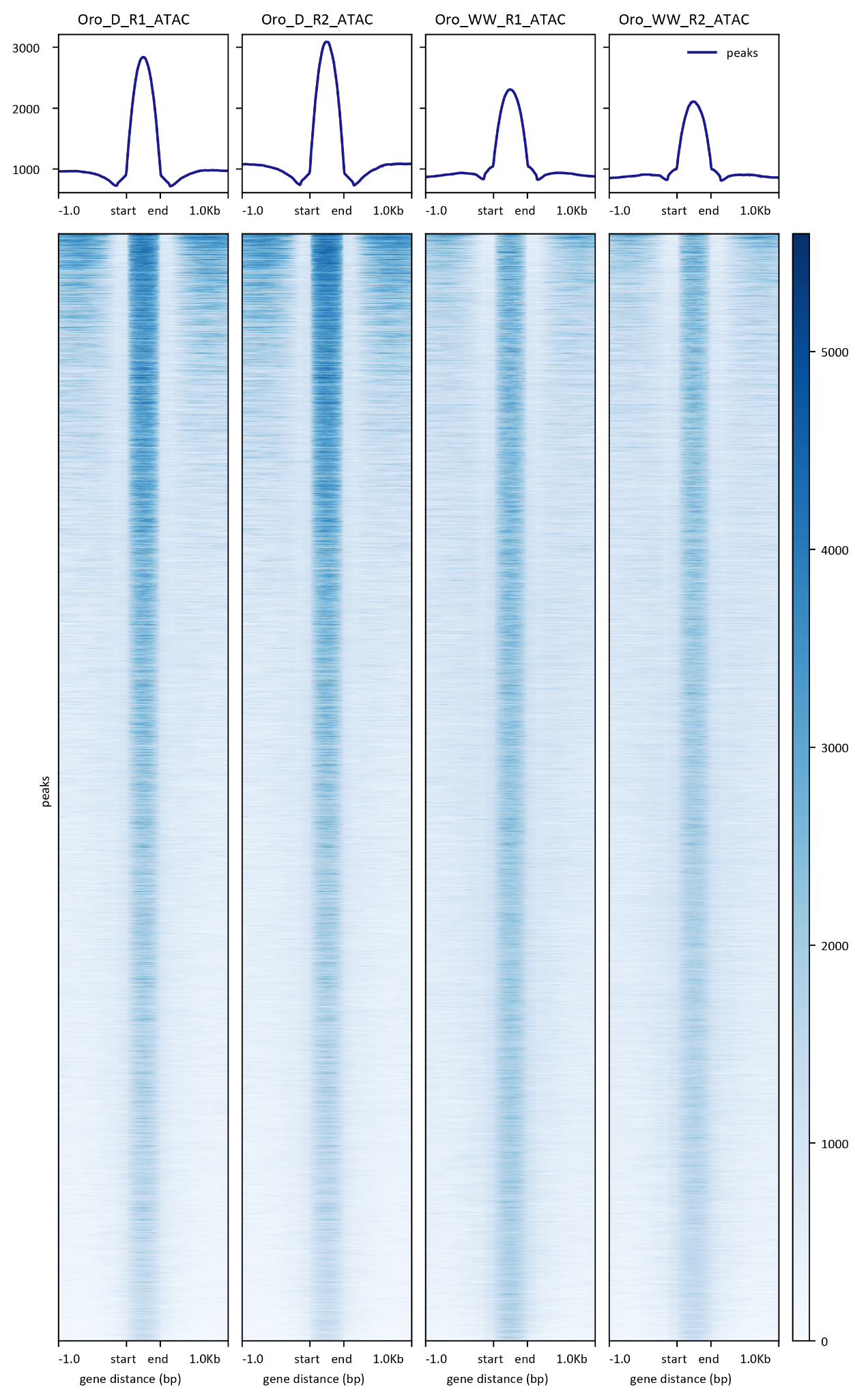


**Fig S3: Scaled plot of accessible chromatin regions near genes in *O. thomaeum*.** Scaled peaks are plotted for the 1 kb upstream, downstream, and genic regions.


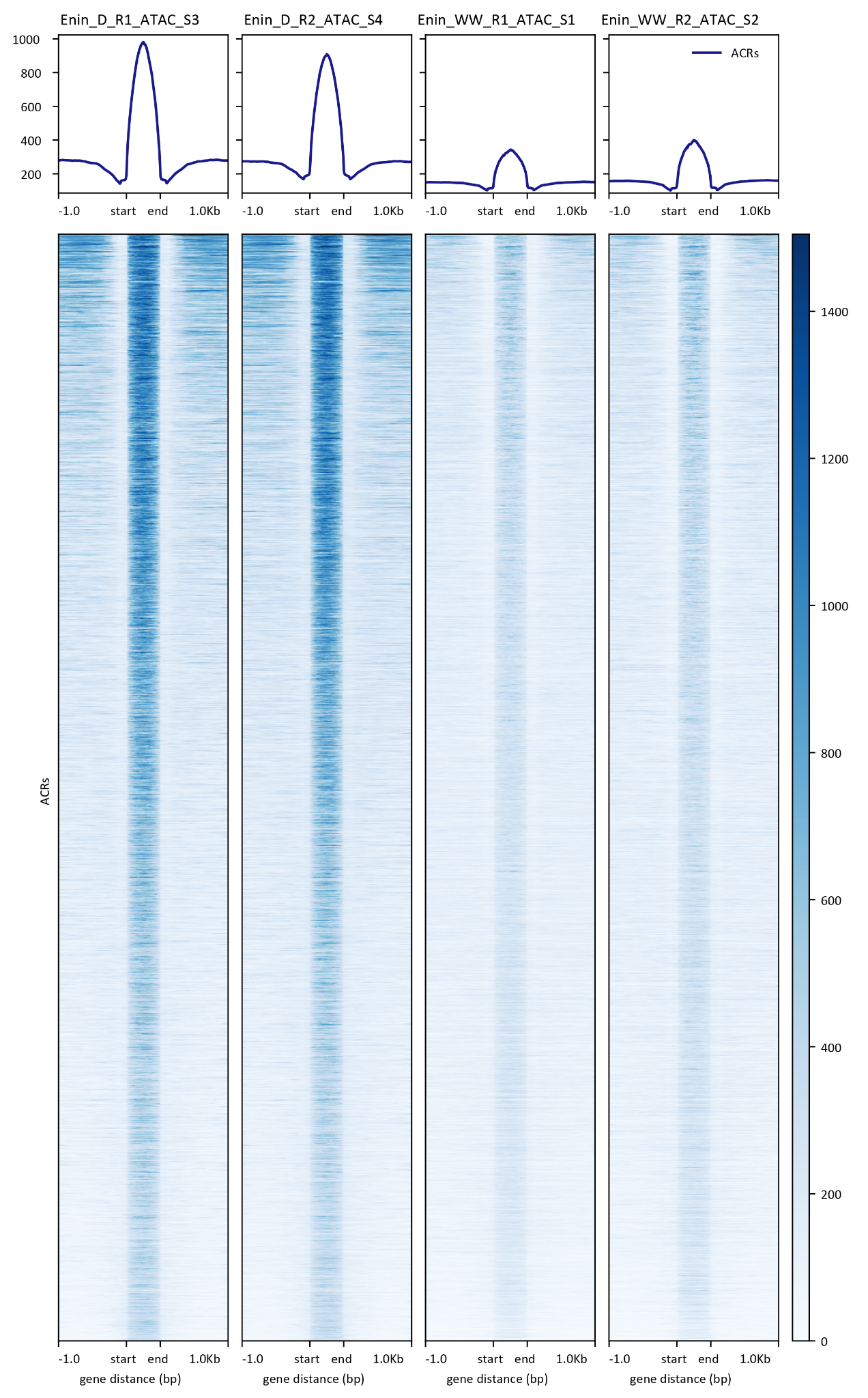


**Fig S4: Scaled plot of open regions and surrounding chromatin area *E. nindensis*.** Scaled peaks are plotted for the 1 kb upstream, downstream, and genic regions.


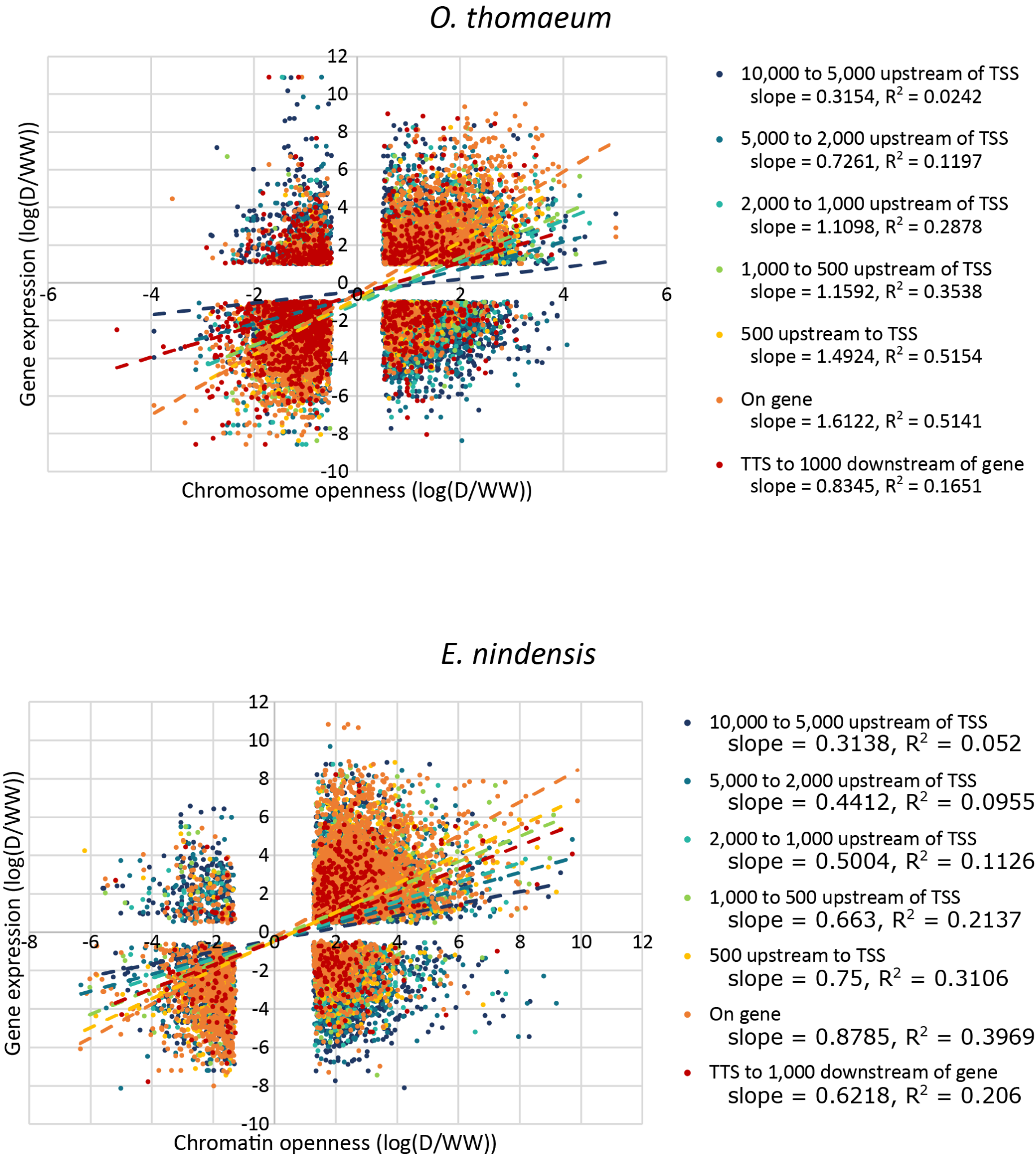


**Fig S5: Correlation between differential gene expression and nearby chromatin openness in (a) *O. thomaeum* and (b) *E. nindensis*.** The differentially expressed genes in desiccation with differentially open chromatin within denoted regions are plotted. Genes in the upper right hand quadrant are more open with higher expression under desiccation. Genes in the lower left hand quadrant have lower expression and less chromatin openness under desiccation.

**
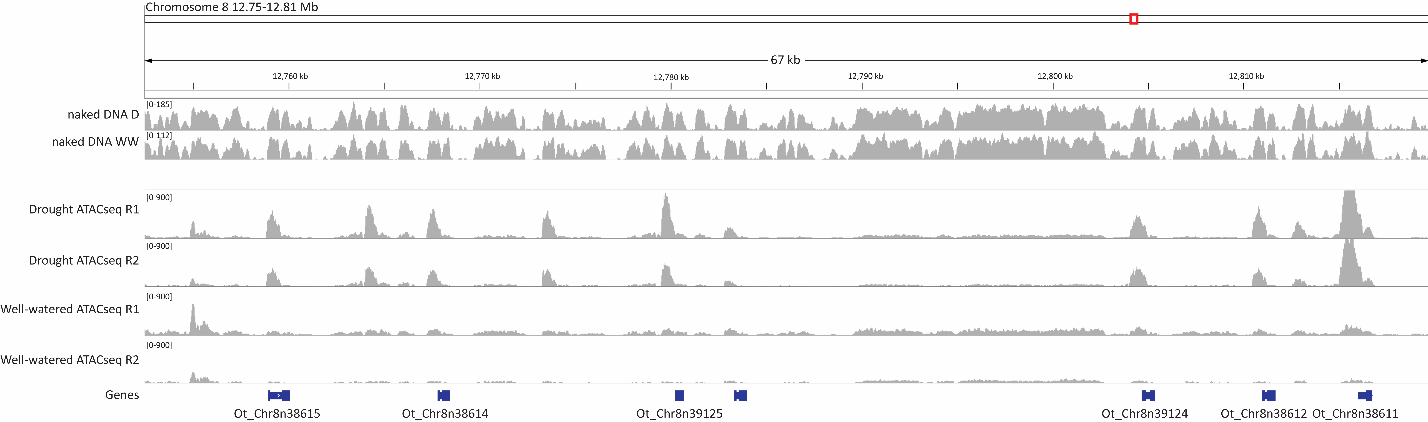
**

**Fig S6: Genome Browser view of ATAC-seq data near the ELIP tandem gene array in *Oropetium thomaeum*.** Aligned bam files for the naked DNA and ATACseq reads and the gene structure tracks are shown.


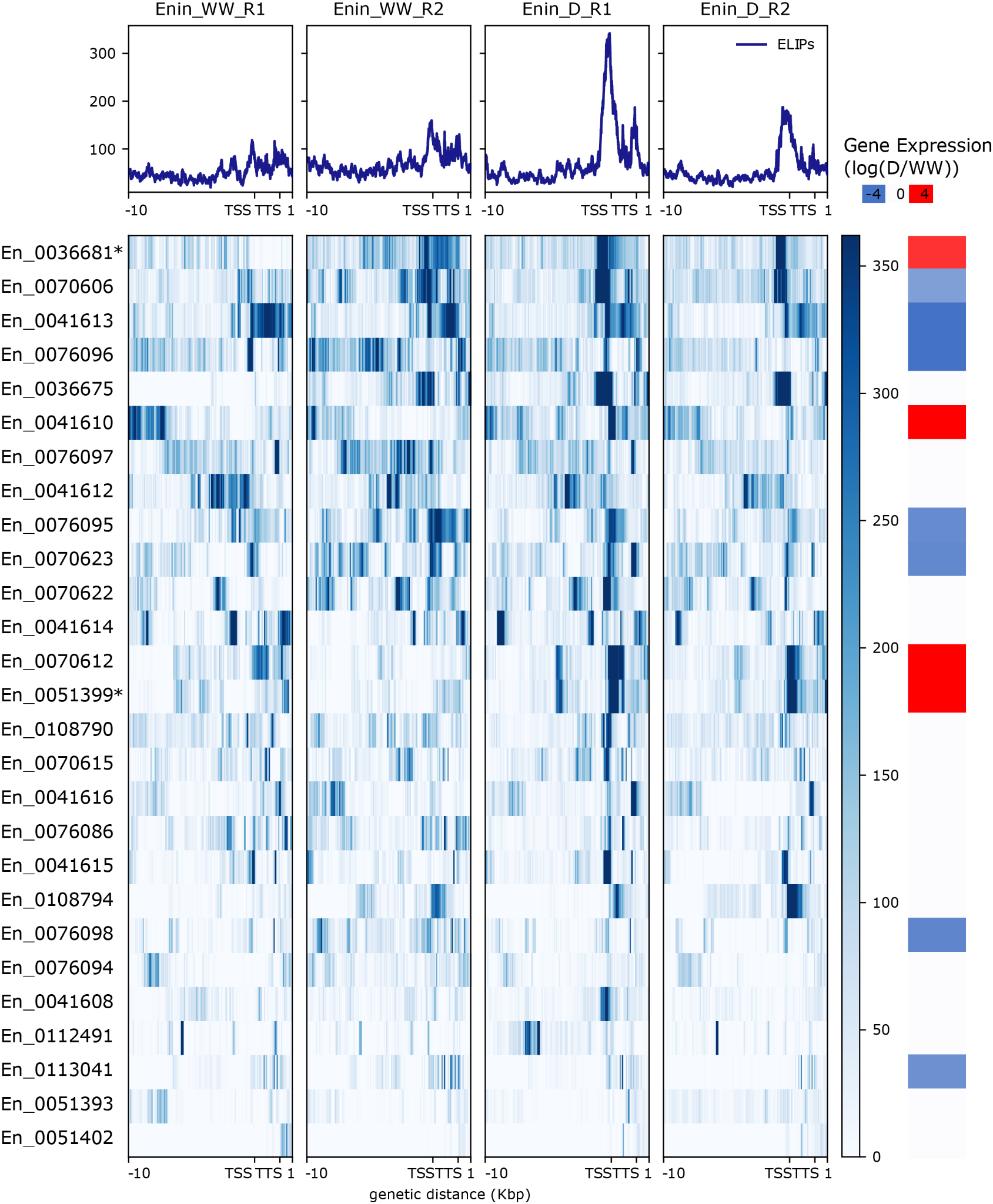


**Fig S7:** **Regulatory dynamics of ELIPs in *E. nindensis.*** Chromatin architecture and expression dynamics of ELIPs in well-watered and desiccated *E. nindensis* samples. The mean mapped read depth of ATACseq reads (in RPKM) is plotted for 10 kb upstream to 1 kb downstream regions for each of the ELIPs in the *O. thomaeum* genome. Log2 transformed RNA expression (in TPM) for each ELIP is shown on the right under well-watered and desiccated conditions.

**Supplemental Tables**

Supplemental Table 1: Differential gene expression in *O. thomaeum*

Supplemental Table 2: Differential gene expression in *E. nindensis*

Supplemental Table 3: Differential chromatin openness in *O. thomaeum*

Supplemental Table 4: Differential chromatin accessibility in *E. nindensis*

Supplemental Table 5: Syntenic differential expression in *O. thomaeum* and *E. nindensis*

Supplemental Table 6: Syntenic chromatin accessibility in *O. thomaeum* and *E. nindensis*

Supplemental Table 7: Gene Ontology Heatmap

Supplemental Table 8: Gene Ontology for different sets of syntenic genes

Supplemental Table 9: Gene Ontology for different sets of genes and chromatin openness
